# Male meiosis, heterochromatin characterization and chromosomal location of rDNA in Microtomus lunifer (Berg, 1900) (Hemiptera: Reduviidae: Hammacerinae)

**DOI:** 10.3897/compcytogen.v5i1.1143

**Published:** 2011-05-05

**Authors:** María Georgina Poggio, María José Bressa, Alba Graciela Papeschi

**Affiliations:** Laboratorio de Citogenética y Evolución, Departamento de Ecología, Genética y Evolución, Facultad de Ciencias Exactas y Naturales, Universidad de Buenos Aires. Int. Güiraldes 2160, C1428EGA, Ciudad Universitaria, Ciudad Autónoma de Buenos Aires, Argentina

**Keywords:** Hemiptera, Reduviidae, Hammacerinae, meiosis, m-chromosomes, evolutionary trends, rDNA-FISH

## Abstract

In the present work, we analysed the male meiosis, the content and distribution of heterochromatin and the number and location of nucleolus organizing regions in *Microtomus lunifer* (Berg, 1900) by means of standard technique, C- and fluorescent bandings, and fluorescent *in situ* hybridization with an 18S rDNA probe. This species is the second one cytogenetically analysed within the Hammacerinae. Its male diploid chromosome number is 31 (2n=28+X1X2Y), including a minute pair of m-chromosomes. The diploid autosomal number and the presence of m-chromosomes are similar to those reported in *Microtomus conspicillaris* (Drury, 1782) (2n=28+XY). However, *Microtomus lunifer* has a multiple sex chromosome system X1X2Y (male) that could have originated by fragmentation of the ancestral X chromosome. Taking into account that *Microtomus conspicillaris* and *Microtomus lunifer* are the only two species within Reduviidae that possess m-chromosomes, the presence of this pair could be a synapomorphy for the species of this genus. C- and fluorescent bandings showed that the amount of heterochromatin in *Microtomus lunifer* was small, and only a small CMA3 bright band was observed in the largest autosomal pair at one terminal region. FISH with the 18S rDNA probe demonstrated that ribosomal genes were terminally placed on the largest autosomal pair. Our present results led us to propose that the location of rDNA genes could be associated with variants of the sex chromosome systems in relation with a kind of the sex chromosome systems within this family. Furthermore, the terminal location of NOR in the largest autosomal pair allowed us to use it as a chromosome marker and, thus, to infer that the kinetic activity of both ends is not a random process, and there is an inversion of this activity.

## Introduction

Reduviidae is the largest family of predaceous land Hemiptera and includes about 6500 species and subspecies in 930 genera and 22 subfamilies. These insects are abundant, occur worldwide, and are voracious predators (thus their name, “assassin bugs”) (Coscarón 1998, Ambrose 1999, Schaefer and Panizzi 2000).

All hemipteran species possess holokinetic chromosomes, i.e. chromosomes without primary constrictions and, hence, without localized centromeres. This order is unique in that the autosomes, m-chromosomes and sex chromosomes have different meiotic behaviours. During mitosis microtubules attach to the entire length of sister chromatids, and at anaphase they segregate parallel to each other and perpendicular to the polar spindle (holokinetic behaviour) (Schrader 1935, Hughes-Schrader and Schrader 1961, White 1973). However, several reports provide evidence that kinetic activity during meiosis is restricted to the chromosome ends where no kinetochore structures are observed, and the chromosomes can be regarded as telokinetic (Motzko and Ruthmann 1984). Both chromosome ends can show kinetic activity in such a way that the chromosome end which was inactive at the first meiotic division become active during the second one (Camacho et al. 1985, Nokkala 1985, Pérez et al. 1997, Cattani et al. 2004, Viera et al. 2009).

As a rule, autosomal bivalents are chiasmatic, whereas sex chromosomes and m-chromosomes are achiasmatic (Ueshima 1979, Manna 1984, Papeschi and Mola 1990, González-García et al. 1996, Suja et al. 2000, Viera et al. 2009). In general, the autosomal bivalents show a single chiasma terminally located (rod bivalents) and orientate at metaphase I with their long axes parallel to the polar axis. During both meiotic anaphases only their ends are able to show kinetic activity leading the chromosome/chromatid segregation to opposite poles (pre-reductional division) (Ueshima 1979, Camacho et al. 1985, Pérez et al. 1997, Viera et al. 2009). Conversely, bivalents with two terminal chiasmata (ring bivalents) orientate with their long axes parallel to equatorial plate and two different behaviours have been described: i) one chiasma releases first, and then one pair of terminal regions becomes free to attach to the spindle and an axial orientation is finally achieved, or ii) alternative sites of kinetic activity become functional (Mola and Papeschi 1993, Papeschi et al. 2003, Viera et al. 2009). On the other hand, the sex chromosomes are achiasmatic and behave as univalents during meiosis I. Most sex chromosomes segregate their chromatids equationally at anaphase I and reductionally at anaphase II (post-reductional division) (Ueshima 1979, Manna 1984, Papeschi and Mola 1990, González-García et al. 1996, Suja et al. 2000, Viera et al. 2009). Finally, the m-chromosomes are generally of the small size, and are usually unpaired and thus achiasmatic during early prophase I. However, previous reports in Coreidae describe the occurrence of regular synapsis of the m-chromosomes (Toscani et al. 2008). At late diakinesis they come close each other, and at metaphase I they are always associated end-to-end, i.e. touch-and-go pairing, forming a pseudobivalent which orientate axially. The first meiotic division is reductional and the second one is equational for the m-chromosomes (Wilson 1909a, Papeschi and Bressa 2006).

Apart from the general characteristics of hemipteran species previously described, the Reduviidae are characterized by a modal diploid number of autosomes of 20 with a range between 10 and 34, and both simple and multiple sex chromosome systems (XY/XX, X0/XX, and XnY/XnXn; male/female) (Ueshima 1979, Manna 1984, Poggio et al. 2007a). Cytogenetic data are currently available for about 152 species belonging to 11 subfamilies; 79 of them belong to Triatominae, 33 to Harpactorinae, 12 to Stenopodainae, and 10 to Peiratinae. The remaining species are evenly distributed among seven other subfamilies: Reduviinae (7 species), Ectrychodiinae (3), Emesinae (3), Phymatinae (2), Bactrodinae (1), Hammacerinae (1), and Saicinae (1) (Poggio et al. 2007a, Kaur et al. 2009, Panzera et al. 2010). Within Hammacerinae, only *Microtomus conspicillaris* (Drury 1782) has been cytogenetically analysed. Its diploid chromosome number is 2n=30 with a sex chromosome system XY/XX and a pair of minute chromosomes denoted as m-chromosomes (Piza 1957).

Furthemore, cytogenetic data for species belonging to Reduviidae point to the presence of C-heterochromatin at terminal regions on a few or all autosomal pairs, and/or on one of the sex chromosomes, whereas the other one is completely heterochromatic (Poggio et al. 2006, Panzera et al. 2010). However, in Triatominae inter- and intraspecific differences in the position, quantity and meiotic behaviour of constitutive heterochromatin have revealed considerable cytogenetic variability (Panzera et al. 2010).

So far, the location of nucleolus organizing regions (NORs) has been analysed in only 14 reduviid species by Ag-NOR, fluorescent banding and/or fluorescent *in situ* hybridization (FISH) with ribosomal DNA (rDNA) probes (18S, 26S or 45S). These results show that in Reduviidae the NOR can be located either at terminal position on one autosomal pair, or on the sex chromosomes. The presence of NORs in both X and Y chromosomes was reported in two species belonging to two different subfamilies (Harpactorinae and Triatominae) (Morielle-Souza and Azeredo-Oliveira 2007, Poggio et al. 2008), and NORs on one autosomal pair plus on one sex chromosome was found in four species, three of them belonging to Triatominae (Morielle-Souza and Azeredo-Oliveira 2007, Bardella et al. 2008, Panzera et al. 2008) and one to Harpactorinae (Poggio et al. 2007b). Of all species analysed, in only two species belonging to Triatominae the NOR regions co-localized with CMA3 bright bands (Severi-Aguiar et al. 2006, Morielle-Souza and Azeredo-Oliveira 2007).

In the present work, we analysed in detail the male meiosis of *Microtomus lunifer* (Berg, 1900) (Hammacerinae) to verify the presence of a pair of m-chromosomes, the content and distribution of heterochromatin by C- and fluorescent bandings, and examined the number and location of NORs by FISH. Lastly, the position of a NOR at the terminal region of the largest autosomal pair allowed us to use it as a chromosome marker and to describe its behaviour during both meiotic divisions.

## Material and methods

### Insects

We used three males of *Microtomus lunifer* from Pampa del Indio, Chaco province (coll. 2008).

### Chromosome preparations

All the analysed specimens were brought alive to the laboratory. The male gonads were dissected in physiological solution. Afterwards, one of the testes was fixed for 15–30 min in freshly prepared Carnoy fixative (ethanol: chloroform: acetic acid, 6:3:1), and was kept at 4ºC in 70% ethanol for meiotic studies. Slides were prepared by the squash technique in a drop of 2% iron acetic haematoxylin following conventional procedures. For C- and fluorescent bandings, and FISH techniques, spread chromosome preparations were made from the other testis as described in Traut (1976). Then the preparations were dehydrated in an ethanol series (70%, 80%, and 96%, 30 sec. each) and stored at −20ºC until use.

### C- and Fluorescent bandings

C- and fluorescent bandings were then applied to spread chromosome preparations to reveal heterochromatin and its base composition. C-banding was performed according to Papeschi (1988). The slides pre-treated for C-banding were stained with 4’,6-diamidino-2-phenylindole (DAPI; Fluka BioChemika, Sigma Aldrich Production GmbH, Buchs, Switzerland) for a better resolution of C-bands.

Fluorescent banding with AT-specific DAPI and GC-specific chromomycin A3 (CMA3; Fluka BioChemika) was carried out as follows: after removal from freezer, the slides were placed immediately into cold 70% ethanol for 2 min. Then, they were transferred through 80% and 100% ethanol, 30 sec each, and air-dried. The slides were submerged in a coplin jar with methanol for two hours. Once dried, they were rinsed with Mc Ilvaine´s buffer pH 7 (0.1 M citric acid, 0.2 M Na2HPO4, in distilled water). Each chromosome preparation was dyed with 75 µl of DAPI solution (0.01 mg/ml, in Mc Ilvaine’s buffer), covered with 24x50 mm transparency cover slides, and kept at room temperature (RT) for 20 min in darkness in a moist chamber. Afterwards, the preparations were rinsed three times using distilled water, Mc Ilvaine’s buffer and distilled water. Then, the slides were dyed with 50 µl of CMA3 solution (0.6 mg/ml, in Mc Ilvaine´s buffer), covered with 24x50 mm transparency cover slide, and incubated at RT for 1 hour in dark in a moist chamber. After this period, the preparations were rinsed again with distilled water, Mc Ilvaine’s buffer and distilled water, and then let them air-dried. The slides were mounted in Antifade based on DABCO (Sigma Aldrich; for composition see Traut (1999)), and covered with 24x40 mm cover glass. The cover glass was sealed with rubber cement, and the slides were stored at 37ºC in dark in a moist chamber three days.

## Fluorescent in situ hybridization with 18S rDNA probes

Unlabelled 18S rDNA probes were generated by polymerase chain reaction (PCR) using universal arthropod primers: forward 5‘-CCTGAGAAACGGCTACCACATC-3’ and reverse 5‘-GAGTCTCGTTCGTTATCGGA-3’ (Whiting 2002). Total genomic DNA of *Dysdercus albofasciatus* Berg, 1978 obtained by standard phenol-chloroform-isoamylalcohol extraction, was used as a template. PCR was done following the procedure described in Fuková et al. (2005). The PCR product showed a single band of about 1000 bp on a 1% agarose gel. The band was cut out from the gel, and the DNA was extracted using a QIAquick Gel Extraction Kit (Quiagen GmbH, Hilden, Germany). The 18S rDNA fragment was re-amplified by PCR and then labelled with biotin-14-dATP by nick translation using a BioNick Labeling System (Invitrogen, Life Technologies Inc., San Diego, CA, USA). FISH with biotinylated 18S rDNA probewas performed essentially following the procedure in Sahara et al. (1999) with several modifications described in Fuková et al. (2005) and in Bressa et al. (2009).

### Analysis of sites of kinetic activity

The location of NOR regions in the largest autosomal pair of *Microtomus lunifer* allowed us to analyse the behaviour of the terminal regions which were kinetically active. The number of cells at metaphase I and metaphase II, in which the kinetically active terminal regions of this autosomal pair were associated to the NOR (Figs 5d, g) or not (Figs 5e, f, were counted. The hypotheses described below were tested using a Chi-squared goodness of fit test.

H01: the kinetic activity of both ends (with/without NOR) at both meiotic divisions is a random process.

H02: the chromosome end that is active during the first meiotic division becomes inactive during the second one and vice versa.

### Microscopy and image processing

Preparations were observed in a Leica DMLB microscope equipped with a Leica DFC350 FX CCD camera and Leica IM50 software, version 4.0 (Leica Microsystems Imaging Solutions Ltd., Cambridge, UK). Black-and-white images of chromosomes were recorded separately for each fluorescent dye. Images were pseudocoloured (light blue for DAPI, green for CMA3, red for Cy3) and processed with an appropriate software.

## Results

### Male chromosome complement and meiosis

**Figure 1.**
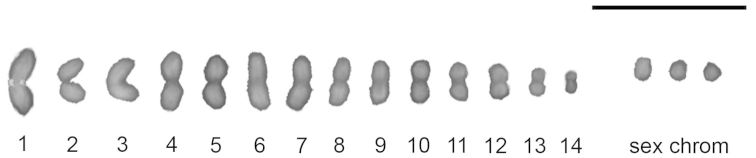
Male meiotic karyotype of *Microtomus lunifer*. Chromosomes are counterstained with DAPI; the largest autosomal pair is recognized by the presence of the rDNA hybridization signals. Bar = 10 μm

**Figure 2a–j.**
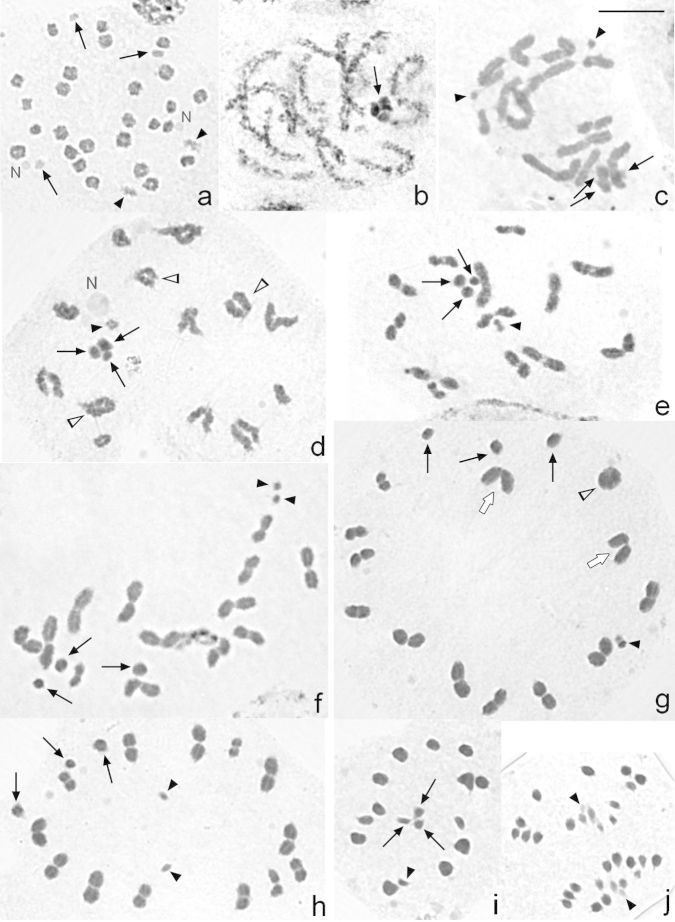
Male meiosis in *Microtomus lunifer*. **a** Spermatogonial prometaphase **b** Late pachytene **c** Diplotene. The smallest chromosome pair is observed as two univalents (black arrowheads) **d** Diplotene. The smallest chromosome pair is as a pseudo-bivalent (black arrowhead) **e** Diakinesis. The smallest chromosome pair is as a pseudo-bivalent (black arrowhead) **f** Diakinesis. The smallest chromosome pair is observed as two univalents (black arrowheads) **g** Metaphase I. The smallest chromosome pair (black arrowhead) formed a pseudo-bivalent and is placed in the bivalent autosomal ring **h** Metaphase I. The smallest chromosome pair lies in the centre of the ring and migrates precociously (black arrowheads) **i** Metaphase II **j** Anaphase II. Black arrows: sex chromosomes. Black arrowheads: smallest chromosome pair. White arrowheads: autosomal bivalents with two chiasmata. White arrows: V-shaped bivalents. N: nucleolus. Chromosomes are stained with 2% iron acetic haematoxylin. Bar = 10 µm

*Microtomus lunifer* possesses a male diploid chromosome number of 31, and its complement comprises 14 autosomal bivalents and a multiple sex chromosome system X1X2Y (Fig. 1). In spermatogonial prometaphase, the sex chromosomes and an autosomal pair are easily recognized because of their small size, whereas the rest of the autosomes cannot be distinguished due to their similar size. An association between a nucleolus and an autosomal pair is observed (Fig. 2a).

At early pachytene, it is not possible to individualize each autosomal bivalent. However, the three sex chromosomes are positively heteropycnotic and lie close to each other forming a pseudo-trivalent. At late pachytene the bivalents continue their condensation, and the sex chromosomes become isopycnotic (Fig. 2b). From diplotene onwards, 13 autosomal bivalents, two univalents and three sex chromosomes are clearly distinguished in some cells (Fig. 2c, f), whereas in other ones 14 autosomal bivalents and three sex chromosomes are also observed (Fig. 2d, e). It can be noticeably seen that the sex chromosomes differ slightly in size (Fig. 2d–f). At metaphase I, the sex univalents lie at the periphery of the ring formed by the autosomal bivalents, and their different size is evident (Fig. 2g, h). At this stage, the smallest chromosome pair does not show any defined position and can be found either being part of the ring (Fig. 2g) or at its centre (Fig. 2h). This smallest pair can be observed migrating precociously in some cells (33 out of 100 cells) (Fig. 2h). At anaphase I, autosomal bivalents divide reductionally, while the sex chromosomes segregate equationally. Therefore, at telophase I two nuclei with 17 chromosomes each (14A+X1X2Y) are observed. Second meiotic division follows immediately after telophase I without an interkinesis stage. At metaphase II, the autosomes dispose at the equatorial plane forming a ring, and in the centre of it the sex chromosomes form a pseudo-trivalent (Fig. 2i). The Y chromosome is orientated towards the spindle pole opposite to that of X1 and X2. At anaphase II, 15 chromosomes migrate to one of the poles (14A+Y) and 16 to the opposite one (14A+X1X2) (Fig. 2j).

There is usually only one chiasma on each autosomal bivalent, which can be terminally or, less frequently, subterminally located, although they can show two chiasmata (Fig. 2d, g). Cells with two ring bivalents are seldom observed, while those with three ring bivalents are even rarer (Fig. 2d). In this species three kinds of bivalents are observed: rod (Fig. 2c–h), ring (Fig. 2d, g) and V-shaped (Fig. 2g) bivalents from diplotene to metaphase I. Mean chiasma frequency in cells at diakinesis-metaphase I is 14.76, being 15 (38.7%, 93 analysed cells) and 14 (68.7%, 99 analysed cells) the most frequent number of chiasmata at diakinesis and at metaphase I, respectively.

### C- and Fluorescent bandings

**Figure 3a–f.**
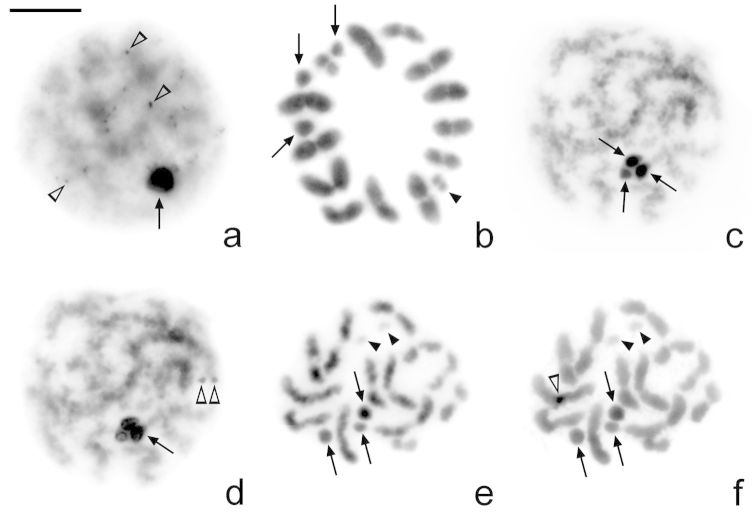
*Microtomus lunifer*. **a–b** C-banding and **c–f** Fluorescent banding: **c, e** DAPI and **d, f** CMA3. **a** Leptotene-zygotene. Very small C-positive dots can be observed in the autosomal chromatin; sex chromosomes are C-positive **b** Metaphase I. No C-positive bands can be detected **c–d** Pachytene **e–f** Diakinesis. No DAPI (**c, e**)and neither CMA3-positive bands (**d, f**) can be detected, except for a small CMA3 bright band in one of the terminal regions of the largest autosomal pair. Arrows: sex chromosomes. Black arrowheads: smallest autosomal pair. White arrowheads: positive dots/bands. Bar = 10 µm

The amount of heterochromatin in *Microtomus lunifer* is small: very small C-positive dots (from 10 to 20) are detected in cells at leptotene-zygotene. At this stage, the sex chromosomes are observed as completely C-positive (Fig. 3a). However, this C-banding pattern can no longer be detected from diplotene onwards (Fig. 3b). All meiotic chromosomes show uniform staining with DAPI (Fig. 3c, e) and CMA3 fluorochromes (Fig. 3d, f), except for the largest autosomal bivalent. A small CMA3 bright band is observed at one of the terminal regions of the largest autosomal pair (Fig. 3d, f). Besides, the smallest pair of chromosomes is both DAPI and CMA3 dull.

### Location of rDNA

**Figure 4a–g.**
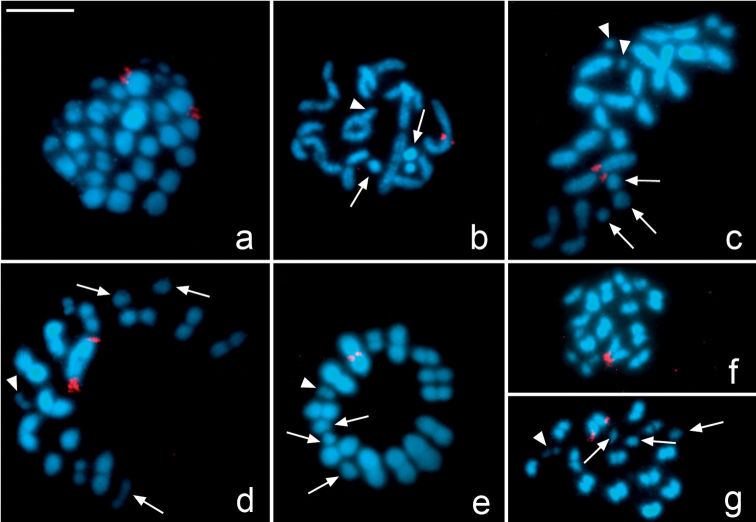
*Microtomus lunifer*. Fluorescent *in situ* hybridization with an 18S rDNA probe. **a** Spermatogonial prometaphase **b** Diplotene **c** Diakinesis **d–e** Metaphase I **f–g** Metaphase II. Hybridization signals in red. Chromosomes are counterstained with DAPI (blue). Arrows: sex chromosomes. White arrowheads: smallest autosomal pair. Bar = 10 µm

In *Microtomus lunifer*, FISH experiments with 18S rDNA probes reveal a single cluster placed at one terminal region of the largest autosomal pair (Fig. 4a–g). In spermatogonial metaphases, it is clearly observed that the hybridization signals are at terminal regions of both sister chromatids of both homologous chromosomes (Fig. 4a). From diplotene onwards, the hybridization signals are detected at one terminal region of the largest autosomal bivalent (Fig. 4b, c). However, both at metaphase I and metaphase II the NOR-autosomal pair shows two different orientations depending on the location of the hybridization signals: the ends with the NOR oriented to the poles (Fig. 4d, g) or the ends without NOR oriented to the poles (Fig. 4e, f).

### Meiotic behaviour and kinetic activity of the NOR-bivalent

**Figure 5a–d.**
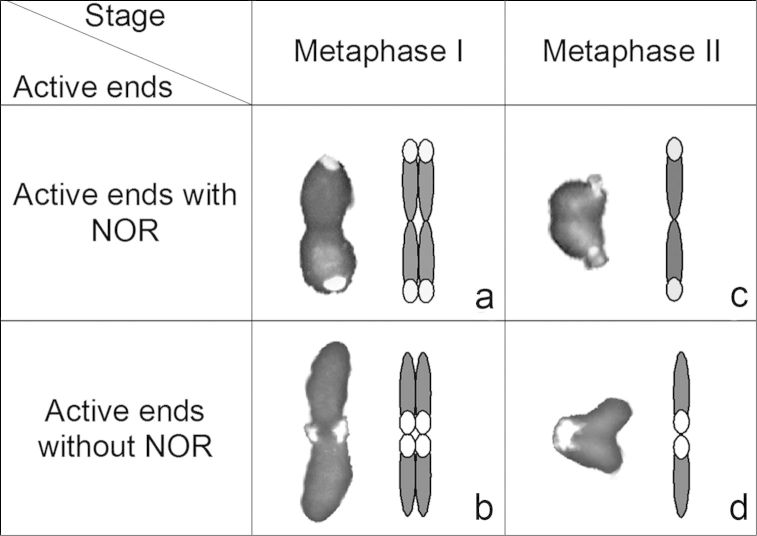
Photos (left) and diagrams (right) illustrating two alternative orientations of the autosomal pair with the NOR: **a–b** at metaphase I and **c–d** metaphase II in *Microtomus lunifer*. Chromosomes: grey; rDNA clusters: white.

In *Microtomus lunifer*,FISH experiments provide a reliable chromosome marker in the NOR-autosome pair to analyse its meiotic behaviour during both meiotic divisions (Figs 4, 5). The presence of a single cluster of rDNA at only one of the ends of each homologous chromosome of the NOR-bivalent allows us to distinguish whether both ends (carrying the NOR or not) take part in the kinetic behaviour of this autosomal pair. At metaphase I, this NOR-bivalent is axially oriented and shows two types of configuration: either the chromosome ends bearing the hybridization signals (Figs 4d, 5a) or the ends that do not bear them are directed towards the poles (Figs 4e, 5b). At metaphase II, the sister chromatids reach an axial orientation and present the same two arrangements: either the chromatid ends carrying the hybridization signals (Figs 4g, 5c) or the other ends that do not carry those (Figs 4f, 5d) are oriented towards the poles.

To test whether the kinetic activity of both ends is a random process at metaphase I and metaphase II, the configurations of the NOR-autosomal bivalent in three individuals were scored. The results demonstrate that at metaphase I the kinetic activity of this NOR-bivalent is restricted to the chromosome ends that do not carry the hybridization signals in 67% of the analysed cells (216 out of 322), whereas in the remaining 33% the kinetic activity occurs at the ends that carry the hybridization signals (Table 1). At metaphase II, however, the kinetic activity is located at the chromatid ends bearing the NOR in 76% of the cells (117 out of 154), and in the remaining cells, at the chromatid ends without it (24% of the cells). Comparing the frequencies of configurations of this NOR-autosome pair between cells at metaphase I and metaphase II, we can observe similar frequencies of cells in which the kinetic activity at metaphase I is restricted to the chromosome ends not carrying the NOR and cells at metaphase II where the kinetic activity is located at the chromatid ends bearing the NOR, and vice versa. Statistical analysis corroborates that: i) the kinetic activity of both ends is not a random process at metaphase I and metaphase II (X2(specimen 1, meta I)=19.5; X2(specimen 1, meta II)=23.5; X2(specimen 2, meta I)=7.71; X2(specimen 2, meta II)=7.11; X2(specimen 3, meta I)=13.16; X2(specimen 3, meta II)=13.53 **>** CV(L=1; α=0.05)=3.84), and ii) the ends that are active during the first meiotic division become inactive during the second one (X2(specimen 1)=3.49; X2(specimen 2)=8.82x10-4; X2(specimen 3)=2.38 < CV(L=1;α=0.05)=3.84).

## Discussion

### Diploid chromosome number, sex chromosome system and chiasma frequency

So far, the cytogenetic analysis of 153 species from Reduviidae reveals a chromosome diploid number that varies from 10 to 34, with both simple and multiple sex chromosome systems (XY/XX, X0/XX, and XnY/XnXn) (Ueshima 1979, Manna 1984, Poggio et al. 2007a, Kaur et al. 2009, Panzera et al. 2010). Within Hammacerinae, *Microtomus lunifer* constitutes the second species cytogenetically analysed, and its diploid autosomal number and the presence of a minute pair of autosomes agree with the previous report in *Microtomus conspicillaris *(2n=30=28+XY/XX) (Piza 1957).

It deserves attention that, even though *Microtomus lunifer* possesses the same diploid autosomal number as *Microtomus conspicillaris*, both species differ in their sex chromosome system; the former has a multiple sex chromosome system X1X2Y (male) whereas the latter presents a simple sex chromosome system XY (male). The most common sex chromosome system in Hemiptera is the simple system XY/XX (male/female). Nevertheless, the other simple system X0/XX, multiple systems (Xn0, XnY, XYn, XnYn) and neo-systems are also reported (Ueshima 1979, Manna 1984, Jacobs 2003, 2004, Papeschi and Bressa 2006, Bressa et al. 2009). In many examples it was described differences in sex chromosomes systems within a genus, and even among species (Pfaler-Collander 1941, Manna 1984, Papeschi 1994, 1996, Bressa et al. 2003). Notwithstandingthe multiple sex chromosome systems are not as common as the simple systems in Hemiptera, the former are especially frequent in Nepidae, Cimicidae and Reduviidae (Ueshima 1979, Poggio et al. 2007a).

It is generally accepted that multiple systems in Hemiptera are the result of fragmentation(s) of the X and/or Y chromosome(s) of an ancestral simple sex chromosome system (Heizer 1950, Hughes-Schrader and Schrader 1961, Ueshima 1979, Manna 1984, Papeschi 1996, Papeschi and Bressa 2006). The holokinetic nature of the hemipteran chromosomes and the achiasmatic behaviour of sex chromosomes during male meiosis are the main facts that support this hypothesis and may account for the variability (Ueshima 1979, Manna 1984, Thomas 1987). In most cases of multiple systems the increase in the number of sex chromosomes is not accompanied by a reduction in the number of autosomes. In most cases, the multiple systems were originated by fragmentation, except in three species of *Acantocephala *(=*Metapodius*) (Laporte, 1833) (Coreidae) (Wilson 1909b) and in *Cimex lectularius* (Linnaeus, 1758) (Cimicidae) (Darlington 1939, Slack 1939, Ueshima 1979, Grozeva et al. 2010) in which the multiple sex chromosome systems could have originated by a non-disjunction. In the present work, the size comparison of the three sex chromosomes of *Microtomus lunifer* with the X and Y chromosomes of *Microtomus conspicillaris*, where the Y is slightly smaller than the X, reveals that the relative size of the X1 plus the X2 does not differ significantly from the size of the single X. Hence, it is suggested that the original X was fragmented into two unequal chromosomes, one larger (X1) than the other (X2). In *Microtomus lunifer*, the male meiotic behaviour of the sex chromosomes was regular, and the new X1 and X2 should have been repaired the broken ends to ensure their stability due to the fragmentation. This hypothesis is also based on that the two X chromosomes of *Microtomus lunifer* are slightly different in size, making it unlikely that this system has arisen by aneuploidy (non-disjunction). Thus, *Microtomus conspicillaris* has the ancestral simple sex chromosome system (XY), and the multiple system X1X2Y of *Microtomus lunifer* might have originated by fragmentation of the ancestral X chromosome.

In Hemiptera, autosomal bivalents are chiasmatic (except in a few families, such as Nabidae, Miridae, Cimicidae; see Nokkala and Grozeva (2000), Poggio et al. (2009)) and present as a rule only one chiasma per bivalent (Ueshima 1979, Manna 1984). Nevertheless, the presence of two terminal chiasmata in large autosomal bivalents has been increasingly reported lately (Camacho et al. 1985, Mola and Papeschi 1993, Bressa et al. 2001, Jacobs and Liebenberg 2001, Rebagliati et al. 2001, Bressa et al. 2002, Jacobs and Groeneveld 2002, Papeschi et al. 2003, Rebagliati et al. 2003, Rebagliati and Mola 2010). On the basis of the meiotic chromosomes behave as telokinetic the ring-shaped bivalents should have some mechanism/s to ensure their attachment to the spindle fibres. Previous reports proposed that both terminal regions and secondary constrictions, or sites beside them, are able of attaching to spindle fibres and of developing kinetic activity (Camacho et al. 1985, Mola and Papeschi 1993, Papeschi et al. 2003). The analysis of chiasma frequency in *Microtomus lunifer *shows that there are from one to three autosomal bivalents with two chiasmata at diakinesis-metaphase I, which increases the expected frequency of 14 to 14.76 (with a range from 14 to 17). Besides, the mean chiasma frequency is higher in diakinesis than in metaphase I. This difference between the two stages may be due to a decrease in the number of autosomal bivalents with two chiasmata that it is consistent with the presence of V-shaped bivalents in metaphase I. Thus, one of the two chiasmata releases firstly, one pair of terminal regions becomes free to attach to the spindle, and the bivalent finally adopts a rod shape.

**Table 1.** Frequencies of cells at metaphase I and metaphase II showing the kinetic activity restricted to the NOR or not NOR ends of the largest autosomal pair.

*Specimen*	*Frequency*	*Metaphase I*		*Metaphase II*	
		*configuration a*	*configuration b*	*configuration c*	*configuration d*
*1*	F1*	24	66	40	7
	f1*	0.27	0.73	0.85	0.15
*2*	F2*	12	30	26	10
	f2*	0.29	0.71	0.72	0.28
*3*	F3*	70	120	51	20
	f3*	0.37	0.63	0.72	0.28

*, Fn = absolute frequency of specimen n; fn = absolute frequency of specimen n

### Evolutionary trends and presence of m-chromosomes

The basal position of Hammacerinae was earlier proposed by Clayton (1990) based on the fact that it is the only subfamily that retained some plesiomorphic characters of the closest sister groups to Reduviidae. All current analyses based on both morphological characters and mitochondrial and nuclear ribosomal genes support the position of Hammacerinae as a sister group of all remaining subfamilies of this family (Weirauch 2008, Weirauch and Munro 2009). Taking into account the cytogenetic characteristics of reduviids, Poggio et al. (2007a) proposed that the ancestral autosomal diploid number for Cimicidae and Reduviidae should be 28, and the evolutionary trends within reduviids should have involved a reduction in autosomal number through fusions and an increase in the number of sex chromosomes through fragmentations (multiple systems). Our present results together with the previous study in *Microtomus conspicillaris* lead us to suggest that Hammacerinae presents the ancestral autosomal diploid number proposed for Reduviidae. Hence, the cytogenetic results support the cladistic analysis of reduviids based on morphological and molecular characters (Weirauch 2008, Weirauch and Munro 2009).

A particular feature of both *Microtomus* species is the presence of a minute chromosome pair with a different meiotic behaviour from that of autosomes and sex chromosomes, the so-called m-chromosomes. Most reports on the behaviour of the m-chromosomes described them as asynaptic and achiasmatic throughout early meiotic prophase after conventional staining squashed spermatocytes. At diakinesis they approach each other, and at metaphase I they are always associated end-to-end (touch-and-go pairing) forming a pseudo-bivalent that segregates reductionally at anaphase I. However, minor modifications of this typical male meiotic behaviour are found among different taxa, particularly with regard to the size, the pycnotic cycle, the meiotic behaviour, and the arrangement at both metaphases I and II (Wilson 1905a, b, 1909a, b, 1911, Nokkala 1986, Suja et al. 2000, Bressa et al. 2005, Toscani et al. 2008). An exception to the lack of synapsis and chiasmata in the m-chromosomes has been described in *Coreus marginatus* (Linnaeus, 1758) (Coreidae). In this coreid bug, some male meiotic cells showed a small synaptonemal complex corresponding to the m-chromosome pair that later appears as a chiasmatic bivalent in diplotene (Nokkala 1986, Suja et al. 2000).

From diplotene onwards, the smallest chromosome pair of *Microtomus lunifer* appeared not only as structures resembling true bivalents (Figs 2d, e; 4b) (Nokkala 1986), but also as two univalents (Figs 2c, f; 3e-f; 4c) (Ueshima 1979, Manna 1984, Papeschi and Bressa 2006). Since this minute chromosome pair could not be recognized until diplotene, it is not possible to assure whether they are asynaptic/achiasmatic or desynaptic. At metaphase I, the m-chromosomes were always observed as a pseudo-bivalent not only lying in the centre of the ring of autosomal bivalents but also forming part of it. Even though the m-chromosomes migrated precociously, this pair of chromosomes as well as the autosomes segregated reductionally during anaphase I.

Taking into account the meiotic behaviour of the m-chromosomes in *Coreus marginatus* and the presence of m-chromosome pair in *Microtomus conspicillaris* our results allow us to suggest that the minute chromosome pair of *Microtomus lunifer* could be considered a pair of m-chromosomes.

Up to now, *Microtomus conspicillaris* and *Microtomus lunifer* are the only two species within Reduviidae that possess a pair of m-chromosomes; thus, the presence of this pair could be a synapomorphy for the species of *Microtomus *Illiger, 1807.

### C- and fluorescent bandings

In Hemiptera early reports on C-positive heterochromatin showed that C-bands are terminally located in some or all the chromosomes. However, interstitial C-positive bands are described in a few species and some of them correspond to secondary constrictions and NORs (Camacho et al. 1985, Panzera et al. 1995, Grozeva and Nokkala 2001, Ituarte and Papeschi 2004, Bressa et al. 2005, Franco et al. 2006, Bressa et al. 2008). The meiotic karyotype of *Microtomus lunifer* is almost devoid of heterochromatin, except for a few dots only detectable at early meiotic prophase in the autosomal chromatin mass.

The use of fluorescent DNA-binding dyes with different specificities allows a better characterization of heterochromatic regions in terms of their relative enrichment with AT or GC base pairs. Most reports referring to heterochromatin characterization on hemipteran species describe C-bands as DAPI bright and CMA3 dull. The presence of a CMA3 bright band was detected in a few species at interstitial or terminal position, either on autosomes or sex chromosomes, and they are generally associated to NORs (González-García et al. 1996, Papeschi et al. 2001, Papeschi et al. 2003, Rebagliati et al. 2003, Cattani et al. 2004, Grozeva et al. 2004, Papeschi and Bressa 2006). In *Microtomus lunifer*, our results of fluorescent bandings show the presence of a small CMA3 bright band in one of the terminal regions of the largest autosomal pair. This CMA3 bright band could represent an NOR (see below).

### Location of rDNA

In Reduviidae the location of NORs was analysed in only 14 species belonging to the subfamilies Harpactorinae (2 species) and Triatominae (12 species) by Ag-NOR, fluorescent banding and/or FISH with rDNA probes (18S, 26S or 45S). The present paper brings the first information about the number and chromosomal location of ribosomal gene clusters in Hammacerinae*.* Using rDNA-FISH we show here that *Microtomus lunifer* has an rDNA cluster, which is located at one terminal region of the largest autosomal pair.

In *Microtomus lunifer *the NOR is associated with a small CMA3 bright band. The results of the fluorescent banding and FISH in this species agree with those described for *Rhodnius pallescens* Barber, 1932 (Morielle-Souza and Azeredo-Oliveira 2007), and *Triatoma vitticeps* (Stål, 1859) (Severi-Aguiar et al. 2006), in which the NOR regions co-localized with a CMA3 positive band and, therefore, the repeating unit of ribosomal is G+C-rich.

Taking into account the data on the number and location of rDNA clusters along with the type of sex chromosome systems in Reduviidae, we can observe different patterns of rDNA distribution. The NOR is generally located at terminal position on the X chromosome, or on both X and Y chromosomes in the species that have XY sex chromosome system. On the other hand, in most cases the NOR is placed at terminal position on an autosomal pair in the species with multiple sex chromosome systems (XnY). Providing that the ancestral male karyotype of Reduviidae had 2n=30=28+XY, the NOR would have been at a terminal region of the sex chromosomes. Thus, a single pair of NOR-autosomes in species with multiple sex systems (XnY) might be due to the ability of NOR to change its number and position (Arnheim et al. 1980, Schubert and Wobus 1985, Zhang and Sang 1999, Shishido et al. 2000, Roy et al. 2005, Datson and Murray 2006, Schubert 2007, Cabrero and Camacho 2008, Bressa et al. 2009, Nguyen et al. 2010).

### Meiotic behaviour and kinetic activity of the NOR-bivalent

In two species of Coreidae, *Carlisis wahlbergi* Stål, 1858and *Camptischium clavipes *(Fabricius, 1803), most crossovers occurred in the distal half of the NOR-bivalent (Fossey and Liebenberg 1995, Cattani et al. 2004). The authors suggested that the NOR could act as a crossover repellent since it could be a hindrance for a recombination event. On the contrary, in the NOR-autosomal bivalent of *Microtomus lunifer*, as well as in the previously analysed species *Nezara viridula* (Linnaeus, 1758) (Pentatomidae) (Camacho et al. 1985), the chiasmata can be formed more frequently at the terminal region near the NOR than in the other one (without NOR). Thus, we could propose that the presence of a NOR does not interfere with the meiotic recombination in this species.

In *Microtomus lunifer *the location of the NOR at one chromosome end was used as a chromosome marker that allowed us to discern both ends, determine whether both terminal regions could be kinetically active and analyse the behaviour of autosomes during both meiotic divisions. The hypotheses which were tested are the followings: i) the kinetic activity of both ends at both meiotic divisions is a random process, and ii) those regions that were active during anaphase I become inactive during anaphase II and vice versa. From our results it can be concluded that both terminal regions are able to develop kinetic activity at first and second meiotic divisions, but the election of the kinetic end is not a random process. In addition, those chromosome ends that show kinetic activity in the first meiotic division are inactive in the second one, and vice versa.

The identification of the factor/s and the mechanism/s involved in the restriction of the kinetic activity to only one chromosome/chromatid end in holokinetic chromosomes of Hemiptera remains unsolved. The presented results here together with previous papers (Pérez et al. 1997, 2000, Viera et al. 2009) allow us to suggest that the euchromatic chromosome ends could present kinetic activity more frequently than the other ends composed of repetitive DNA sequences, i.e. blocks of heterochromatin or ribosomal genes, at metaphase I, and vice versa at metaphase II. However, it will be necessary to analyse more hemipteran species to elucidate the factor/s and the mechanism/s that influence the determination of those ends kinetically active in their holokinetic chromosomes.

In summary, the analysis of meiosis, the determination of the distribution, number and location of heterochromatin blocks and rDNA loci could be useful for the taxonomic identification of species, the analysis of karyotype evolution, and for a better knowledge of chromosome structure and organization.
